# Explainable machine learning to predict postoperative ileus after radical cystectomy: an 11-year real-world cohort

**DOI:** 10.3389/frai.2025.1678292

**Published:** 2025-11-25

**Authors:** Xiaoping Chen, Guolong Chen, Zongxin Zheng, Huiming Lu, Yuyi Luo, Lu Man, Mengxiao Jiang

**Affiliations:** 1 Urology Department, Sun Yat-sen University Cancer Center, Guangzhou, China; 2 State Key Laboratory of Oncology in South China, Guangzhou, China; 3 Guangdong Provincial Clinical Research Center for Cancer, Guangzhou, China

**Keywords:** machine learning, postoperative ileus, radical cystectomy, risk stratification, predictive modelling, Shapley additive explanations

## Abstract

**Background:**

Post-operative ileus (POI) is a frequent complication after radical cystectomy (RC). Conventional scores capture only linear relations and have limited accuracy. Interpretable machine learning (ML) may improve early risk stratification.

**Methods:**

In a single-centre real-world cohort (*n* = 1,062, 2013–2023), POI was defined by ≥2 standard clinical–radiological criteria. We extracted pre-operative comorbidities/medications, operative factors (approach, urinary diversion, lymph-node dissection, fluids, blood loss, nasogastric-tube placement) and first-day laboratory indices. After LASSO selection, five ML models were trained/validated on a stratified split; discrimination (AUC), accuracy, precision, recall and Brier score were compared. SHAP delivered global and patient-level explanations.

**Results:**

POI occurred in 28.9%. The back-propagation neural network performed best (AUC 0.828; accuracy 78.4%; Brier 0.143). Intra-operative nasogastric-tube placement and surgical approach dominated feature attribution, followed by medication history, lymph-node dissection, lymphocyte count and C-reactive protein. SHAP clarified feature effects and enabled interpretable, case-level risk summaries.

**Conclusion:**

An interpretable ML model based on routinely captured peri-operative variables accurately stratifies RC patients at risk for POI as early as postoperative day 0, outperforming existing nomograms and highlighting modifiable factors. Embedding this tool into electronic-health-record workflows could enable real-time alerts and risk-adapted management. Prospective multicentre validation is warranted.

## Introduction

In 2020, approximately 573,000 new cases of bladder cancer were reported globally, accounting for approximately 3.0% of all cancers, ranking tenth worldwide (Wéber et al., 2024). In China, the incidence of bladder cancer was 7.03 per 100,000 people in 2019, showing an upward trend since 1990 (Xiang et al., 2022). Radical cystectomy (RC) remains the gold standard treatment for muscle-invasive bladder cancer and is used to select high-risk, non-muscle-invasive tumors. Despite advances in perioperative care, postoperative ileus (POI) continues to complicate 18–30% of cases, contributing to prolonged length of stay, higher readmission rates, and increased healthcare costs (Grilo et al., 2024). POI typically develops 3–5 days after surgery and is characterised by abdominal distension and delayed return of bowel function (Bragg et al., 2015; Nutt et al., 2018).

Understanding and management of POI remains limited because of its multifactorial etiology involving surgical techniques, patient characteristics, and early postoperative management (Lou et al., 2022). The multifactorial etiology of POI involves surgical methods, patient characteristics, and postoperative protocols. Previous studies have identified risk factors such as elevated preoperative creatinine, hypoalbuminemia, older age, lower BMI, and specific urinary diversion types (Meng et al., 2015; Tan et al., 2019; Xue et al., 2021; Zhang et al., 2022; Sun et al., 2024). For example, Xue et al. (2021) proposed a point-based risk scale for POI in patients. However, these tools are often derived from highly selected cohorts or controlled trial environments, and may not reflect the heterogeneity of real-world practice, where patient comorbidities, surgeon preference, and resource availability vary widely across centers.

Machine-learning (ML) algorithms can learn complex, nonlinear relationships from high-dimensional data and frequently outperform traditional regression models in terms of predictive accuracy (Wang et al., 2023). By focusing on routinely captured preoperative data, intraoperative details, and early (<24 h) postoperative laboratory indices, an ML model can stratify POI risk on postoperative day 0–1, giving clinicians a practical window to intensify monitoring, optimize fluid therapy, and initiate targeted gastrointestinal management. Interpretable techniques, such as Shapley additive explanations (SHAP), further enable clinicians to visualize variable importance and gain biological insight (Greener et al., 2022; Zhou et al., 2024). Capturing real-world variability while providing actionable, time-critical information can reduce patient burden and improve the quality of life (Dong et al., 2021).

Therefore, the present study aimed to develop and internally validate an explainable ML model for predicting POI in bladder cancer patients undergoing RC using an 11-year single-center real-world cohort. We hypothesized that an interpretable ML framework would deliver higher predictive performance than traditional methods and provide clinically actionable insight for perioperative teams to prioritize timely, targeted management.

## Methods

### Setting and participants

This retrospective study included patients with RC from a single center (Sun Yat-sen University Cancer Center) between January 2013 and December 2023. The inclusion criteria were adult patients (≥18 years of age) who underwent radical total vesicectomy for muscle-invasive bladder cancer via any surgical method (including open, laparoscopic, or robot-assisted procedures) and had complete clinical data, including routine blood tests and history records. Patients who underwent more than two surgeries during the same hospital stay, had severe psychiatric disorders or cognitive impairments, or had intestinal obstruction due to other diseases were excluded.

This study was conducted in accordance with the principles of the Declaration of Helsinki. Owing to the observational nature of the study and all data collected from medical records, the study was approved by the Ethics Committee of Sun Yat-sen University Cancer Center (Ethics Approval Number: B202400801), and written informed consent was waived.

### Data collection

Clinical data and preoperative and intraoperative factors associated with POI were extracted from electronic medical records. The collected variables comprised demographics (sex, age, and body mass index), medical history, including previous gastrointestinal conditions (irritable bowel syndrome, chronic constipation, inflammatory bowel disease, or prior major abdominal/pelvic surgery), hypertension, diabetes, and preoperative medication history (*μ*-opioid agonists, anticholinergics, systemic corticosteroids, or prokinetic agents used within 30 days before surgery).

Surgical details captured included the operation method (open, conventional laparoscopy, or robot-assisted), type of urinary diversion, concurrent lymph node dissection, intraoperative metrics (crystalloid/colloid volume, red-blood-cell transfusion volume, blood loss, and nasogastric-tube placement), and early postoperative laboratory results obtained within 24 h (hemoglobin, platelet count, white blood cell and differential counts, C-reactive protein, creatinine, and albumin). Twenty-four-hour pelvic-drainage volume was also recorded.

All predictors were selected because they are routinely available and have been reported to be significant correlates of POI in previous studies.

### Definition of POI

POI was diagnosed if at least two of the following criteria were met: (Iskander, 2024) (1) absence of return of bowel function (e.g., bowel movement, flatus, defecation) on postoperative day 5 or later; (2) postoperative emesis or abdominal distension necessitating cessation of oral intake; and (3) multiple air-fluid levels observed on computed tomography or X-ray scans. Two researchers independently diagnosed POI.

## Machine learning development process

### Data preprocessing

One-hot encoding was applied to categorical variables, and numerical features were scaled using min-max normalization. The dependent variable ileus was used as the stratification target. Patients were randomly divided into training and test sets at a 70:30 ratio using stratified sampling based on the presence or absence of POI to ensure outcome balance and reduce selection bias. There were no missing data in this dataset.

### Algorithm training and validation

The dataset was randomly divided into a training set (70%) and a test set (30%) using stratified sampling based on the POI status. The training set was used for model development and hyperparameter tuning via five-fold cross validation. The final model performance was evaluated using a test set to simulate the real-world clinical applicability.

Multivariate analysis employed LASSO regression for variable selection, retaining features with non-zero coefficients at the optimal *λ* that maximized the AUC. These selected features were used to train five machine learning models: Support Vector Machine (SVM), Random Forest (RF), Backpropagation Neural Network (BPNN), Extreme Gradient Boosting (XGBoost), and K-Nearest Neighbors (KNN).

Model performance was evaluated using the test set by calculating the accuracy, precision, sensitivity, specificity, false-positive rate, and false-negative rate. Receiver operating characteristic (ROC) curves and confusion matrices were generated. Model interpretability was assessed using Shapley additive explanation (SHAP) analysis.

For the BPNN model, the architecture included an input layer (corresponding to the selected features), a single hidden layer with 10 neurons using ReLU activation, and an output layer with a sigmoid activation function for binary classification. The hyperparameters were tuned via five-fold cross-validation using a grid search. Early stopping was applied to prevent overfitting and to enhance the reproducibility and robustness of the model.

To ensure transparency and reproducibility, model hyperparameters were optimized using grid search with five-fold cross-validation. The optimized configurations included the number of trees and maximum depth for RF, learning rate and depth for XGBoost, kernel and penalty parameter for SVM, and the number of neighbors for KNN. For the BPNN, a single hidden layer architecture with ReLU activation was employed. All random processes were controlled with random_state = 42.

### Statistical analysis

The data analysis and model construction in this study are conducted using R software (version 4.3.2) and Python software (version 3.11.1). Continuous variables were summarized as mean ± standard deviation (x ± *s*) for normally distributed data or as median and interquartile range (IQR) for skewed distributions, as determined by the Shapiro–Wilk test. Categorical variables are presented as counts and percentages. Group comparisons were conducted using the independent samples t-test for normally distributed continuous variables and the Mann–Whitney *U* test for non-normally distributed data. Chi-square tests were used to analyze categorical data. For model evaluation, accuracy, sensitivity, precision, *F*1 score, and AUC were calculated. Statistical significance was defined as a two-tailed *p* < 0.05.

## Results

### Baseline characteristics

Between January 2013 and December 2023, 1,062 patients were included in this study, with ileus occurring in 307 patients (28.9%). The median age of the total patient population was 63 years, and 85.2% of the patients were male. A comparison of the demographic and clinical characteristics of the patients with and without ileus is shown in Table 1.

**Table 1:** Comparison of demographic and clinical characteristics between patients with and without ileus.

Variables	No Ileus (*n* = 755)	Ileus (*n* = 307)	*p* value
Demographics			
Age (years), median (IQR)	63.00 (56.00–70.00)	63.00 (57.00–69.50)	0.806
BMI (kg/m^2^), median (IQR)	22.93 (21.09–24.90)	22.58 (20.37–24.50)	**0.032**
Gender, *n* (%)			0.866
Male	642 (85.0)	263 (85.7)	
Female	113 (15.0)	44 (14.3)	
Comorbidities & history			
Hypertension, *n* (%)			0.087
No	553 (73.2)	241 (78.5)	
Yes	202 (26.8)	66 (21.5)	
Diabetes, *n* (%)			0.124
No	662 (87.7)	280 (91.2)	
Yes	93 (12.3)	27 (8.8)	
Previous GI conditions, *n* (%)			0.477
No	727 (96.3)	292 (95.1)	
Yes	28 (3.7)	15 (4.9)	
Medication history, *n* (%)			**<0.001**
No	224 (29.7)	151 (49.2)	
Yes	531 (70.3)	156 (50.8)	
Surgery-related			
Operation method, *n* (%)			**<0.001**
Robot-assisted RC	185 (24.5)	17 (5.5)	
Laparoscopic RC	560 (74.2)	289 (94.1)	
Open RC	10 (1.3)	1 (0.3)	
Urinary diversion, *n* (%)			0.910
Ileal conduit	548 (72.6)	223 (72.6)	
Orthotopic neobladder	179 (23.7)	71 (23.1)	
Cutaneous ureterostomy	28 (3.7)	13 (4.2)	
Lymph-node dissection, *n* (%)			**<0.001**
No	155 (20.5)	119 (38.8)	
Yes	600 (79.5)	188 (61.2)	
Hysterectomize, *n* (%)			0.310
No	690 (91.4)	287 (93.5)	
Yes	65 (8.6)	20 (6.5)	
Urethrectomy, *n* (%)			0.081
No	696 (92.2)	272 (88.6)	
Yes	59 (7.8)	35 (11.4)	
Intra-op NGT placement, *n* (%)			**<0.001**
No	205 (27.2)	6 (2.0)	
Yes	550 (72.8)	301 (98.0)	
Intraoperative fluid intake (mL), median (IQR)	2,500 (2000–3,000)	2,500 (2000–3,200)	**<0.001**
Blood loss (mL), median (IQR)	200 (100–400)	200 (100–500)	0.151
Early post-op labs			
WBC (10^9^/L), median (IQR)	6.79 (5.67–8.36)	6.94 (5.74–8.75)	0.224
Lymphocytes (10^9^/L), median (IQR)	1.72 (1.37–2.12)	1.60 (1.30–2.00)	**0.030**
Neutrophils (10^9^/L), median (IQR)	4.20 (3.20–5.42)	4.40 (3.56–5.79)	**0.011**
Hb (g/L), median (IQR)	126.40 (111.50–140.00)	126 (113.00–140.00)	0.573
PLT# (10^9^/L), median (IQR)	248 (203.00–307.00)	245 (201.50–307.50)	0.302
CRP (mg/L), median (IQR)	2.19 (0.99–6.24)	2.39 (1.19–8.20)	**0.041**
CRE (mmol/L), median (IQR)	83.20 (70.55–101.25)	83 (70.25–100.70)	0.733
ALB (g/L), median (IQR)	40.60 (37.80–43.20)	40 (37.50–42.60)	0.065
Pelvic drainage (mL), median (IQR)	250 (130–380)	250 (120–400)	0.961

Continuous variables are expressed as median (IQR); categorical variables as *n* (% within column). Differences were assessed with Mann–Whitney *U* tests (continuous) or *χ*^2^/Fisher exact tests (categorical); bold denotes *p* < 0.05. BMI, body-mass index; NGT, nasogastric tube; CRP, C-reactive protein. Medication history refers to *μ*-opioid agonists, anticholinergics, corticosteroids or pro-kinetic agents used ≤30 days pre-operatively. Previous GI conditions include IBS, chronic constipation, inflammatory bowel disease or prior major abdominal/pelvic surgery.

### Feature selection

**Figure 1:**
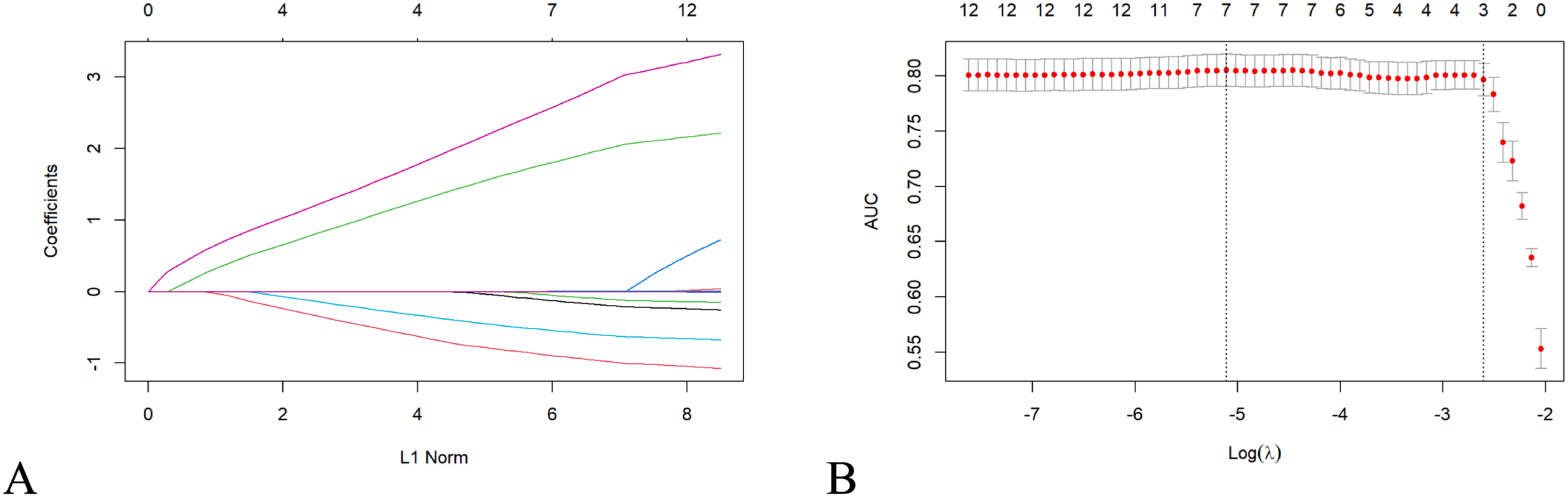
LASSO regression selects clinical features. LASSO regression analysis selecting clinical features for model training. **(A)** Regression coefficients of selected predictors. **(B)** Cross-validation plot indicating the optimal *λ* value where AUC is maximized.

For feature selection, LASSO regression analysis was applied to the 11 variables identified as significant in the univariate analysis. This method, which regularizes coefficients to prevent overfitting, selects seven variables with non-zero coefficients, indicating their importance in predicting POI risk. These variables included operation history, medication history, operation method, lymphatic dissection, gastric tube placement, lymphocyte count, and C-reactive protein (CRP) level. The selected features are shown in Figure 1.

### Performance evaluation of machine learning algorithms

Among the 1,062 patients, 743 were randomly allocated to the training set and 319 to the test set in a ratio of 70:30. There were no statistically significant differences in baseline clinical features between the training and verification groups (*p* > 0.05) (Table 2).

**Table 2:** Training set vs. test set characteristics after variable trimming.

Variables	Training (*n* = 743)	Test (*n* = 319)	*p* value
Demographics			
Age (years), median (IQR)	63 (56–70)	63 (56–70)	0.860
BMI (kg/m^2^), median (IQR)	22.85 (20.76–24.83)	22.93 (21.10–24.80)	0.433
Gender (male), *n* (%)	638 (85.9)	267 (83.7)	0.413
Comorbidities & history			
Hypertension (yes), *n* (%)	188 (25.3)	80 (25.1)	1.000
Diabetes (yes), *n* (%)	85 (11.4)	35 (11.0)	0.908
Previous GI conditions (yes), *n* (%)	31 (4.2)	12 (3.8)	0.888
Medication history (yes), *n* (%)	485 (65.3)	202 (63.3)	0.589
Surgery-related			
Operation method, *n* (%)			0.678
Robot-assisted RC	140 (18.8)	62 (19.4)	
Laparoscopic RC	594 (80.0)	255 (79.9)	
Open RC	9 (1.2)	2 (0.6)	
Urinary diversion, *n* (%)			0.270
Ileal conduit	529 (71.2)	242 (75.9)	
Orthotopic neobladder	185 (24.9)	65 (20.4)	
Cutaneous ureterostomy	29 (3.9)	12 (3.8)	
Lymph-node dissection, *n* (%)	553 (74.4%)	235 (73.7%)	0.855
Hysterectomize, *n* (%)	57 (7.7%)	28 (8.8%)	0.627
Urethrectomy, *n* (%)	71 (9.6%)	23 (7.2%)	0.264
Intra-op NGT placement (yes), *n* (%)	599 (80.6)	252 (79.0)	0.601
Intraoperative fluid intake (ml), median (IQR)	2,500 (2000–3,000)	2,500 (2000–3,000)	0.567
Blood loss (mL), median (IQR)	200 (100–400)	200 (100–400)	0.532
Early post-op labs			
WBC (109/L), median (IQR)	6.85 (5.71–8.43)	6.79 (5.59–8.54)	0.577
Lymphocytes (109/L), median (IQR)	1.70 (1.34–2.08)	1.70 (1.31–2.15)	0.937
Neutrophils (109/L), median (IQR)	4.29 (3.40–5.50)	4.20 (3.23–5.53)	0.507
Hb (g/L), median (IQR)	126 (112–140)	127 (112–140)	0.960
PLT# (109/L), median (IQR)	247 (203–306.70)	247 (202.50–309.50)	0.855
CRP (mg/L), median (IQR)	2.19 (0.99–6.40)	2.40 (1.15–7.02)	0.202
CRE (mmol/L), median (IQR)	83.50 (71.65–101.15)	81.60 (67.70–100.90)	0.313
ALB (g/L), median (IQR)	40.30 (37.90–42.90)	40.40 (37.60–43.00)	0.797
Pelvic drainage (ml), median (IQR)	250 (130–380)	250 (125–380)	0.395

Continuous variables are median (IQR); categorical variables are *n* (% within column). Group differences were tested with Mann–Whitney *U* (continuous) or *χ*^2^ (categorical) as appropriate. Medication history = *μ*-opioid agonists, anticholinergics, corticosteroids or pro-kinetic agents ≤30 days pre-operatively. Previous GI conditions include IBS, chronic constipation, IBD or prior major abdominal/pelvic surgery.

**Figure 2:**
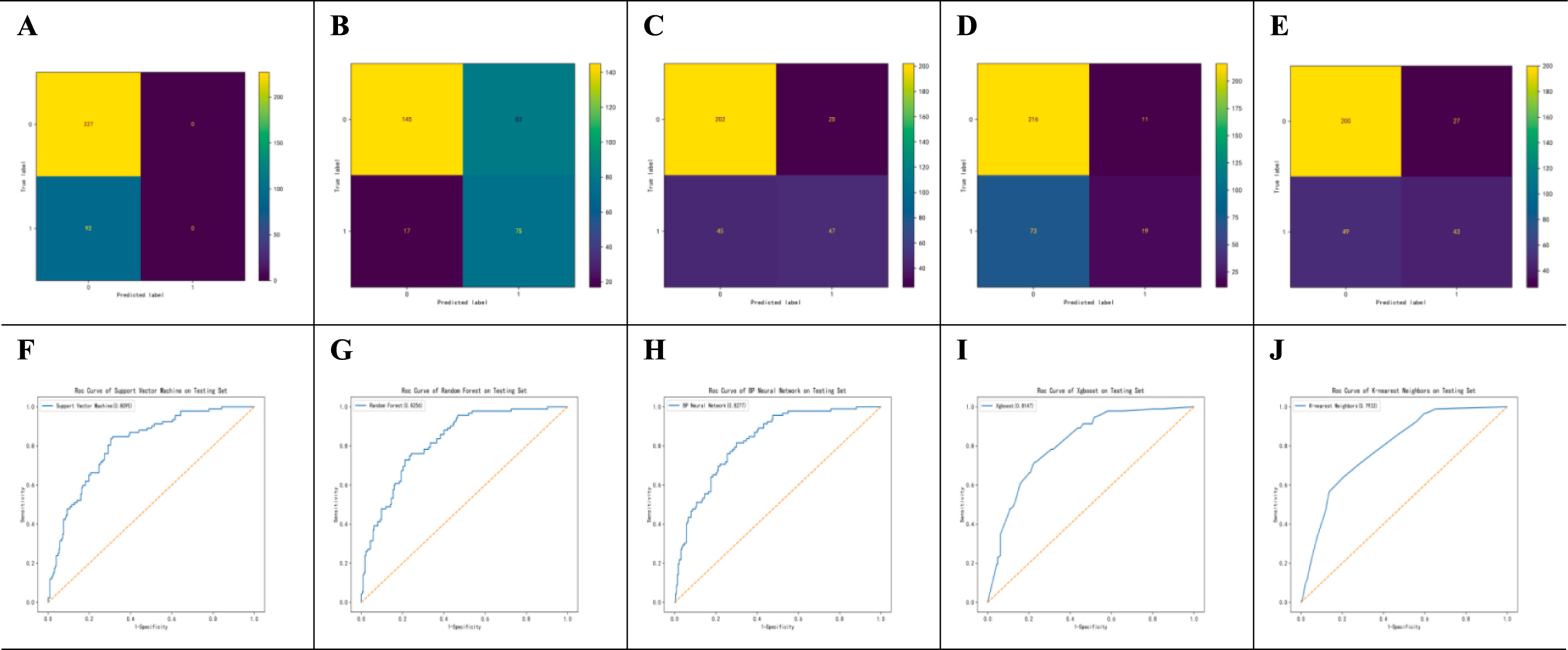
Performance Evaluation of SVM, RF, BPNN, AdaBoost, and KNN Algorithms. Performance evaluation of machine learning models for POI prediction. **(A–E)** Confusion matrices for SVM, RF, BPNN, XGBoost, and KNN models. **(F–J)** ROC curves for SVM, RF, BPNN, XGBoost, and KNN models on the test set. The BPNN model achieved the highest AUC (0.828).

**Figure 3:**
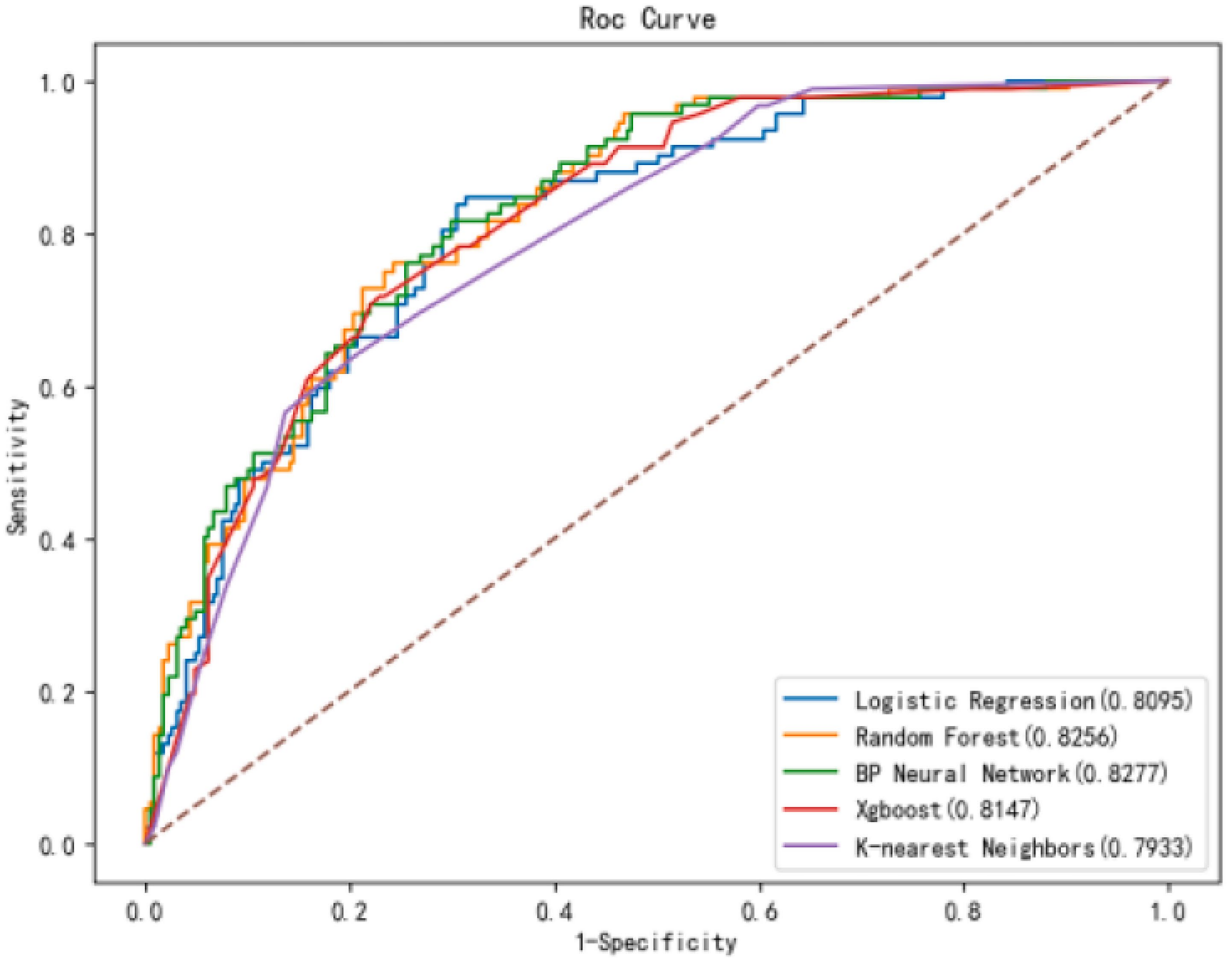
ROC curves of 5 models in the test set. Comparison of ROC curves for all models in the test set. The BPNN model demonstrated superior discriminative ability with an AUC of 0.828, surpassing other models.

As shown in Figure 2, the confusion matrices indicate that Random Forest (RF) performs well in identifying positive cases, whereas the (SVM) shows high accuracy in detecting negative cases. The ROC curves in Figure 2 demonstrate that the (BPNN) achieved the highest AUC (0.828), followed closely by RF (0.8256), SVM, XGBoost, and KNN, with AUC values of 0.806, 0.794, and 0.804, respectively. Figure 3 further compares the ROC curves of the models on the test set, where the BPNN maintained the highest AUC at 0.8277, suggesting robust predictive capability, whereas RF and XGBoost demonstrated competitive performance.

Table 3 provides detailed metrics for each model, highlighting that the BPNN had the highest accuracy (78.4%) and AUC (0.828). RF, with an AUC of 0.818, achieved the highest sensitivity (82.6%), making it advantageous for high-sensitivity scenarios. SVM showed a balanced performance with an AUC of 0.806 and an accuracy of 77.1%. Although XGBoost has a relatively high precision (0.667), its sensitivity is the lowest (17.4%), impacting its overall predictive power. In summary, the BPNN and RF models exhibit strong overall predictive performance, with the BPNN slightly leading to accuracy and AUC, whereas RF is preferable when sensitivity is prioritized.

**Table 3:** Performance of each model for prediction.

Model	Accuracy (%)	Precision	Sensitivity (%)	*F*1	AUC^e^ (%)
SVM^a^	0.771	0.634	0.489	0.552	0.806
RF^b^	0.705	0.494	0.826	0.618	0.818
BPNN^c^	0.784	0.689	0.457	0.549	0.828
XGBoost	0.737	0.667	0.174	0.276	0.794
KNN^d^	0.752	0.582	0.500	0.538	0.804

SVM, Support Vector Machine; RF, Random Forest; BPNN, BP Neural Network; KNN, K-Nearest Neighbors; AUC, Area Under the Curve. Metrics include model Accuracy, Precision, Sensitivity, *F*1 Score, and AUC. The BPNN model showed the highest AUC (0.828), indicating superior predictive performance for POI.

### Feature importance in POI

**Figure 4:**
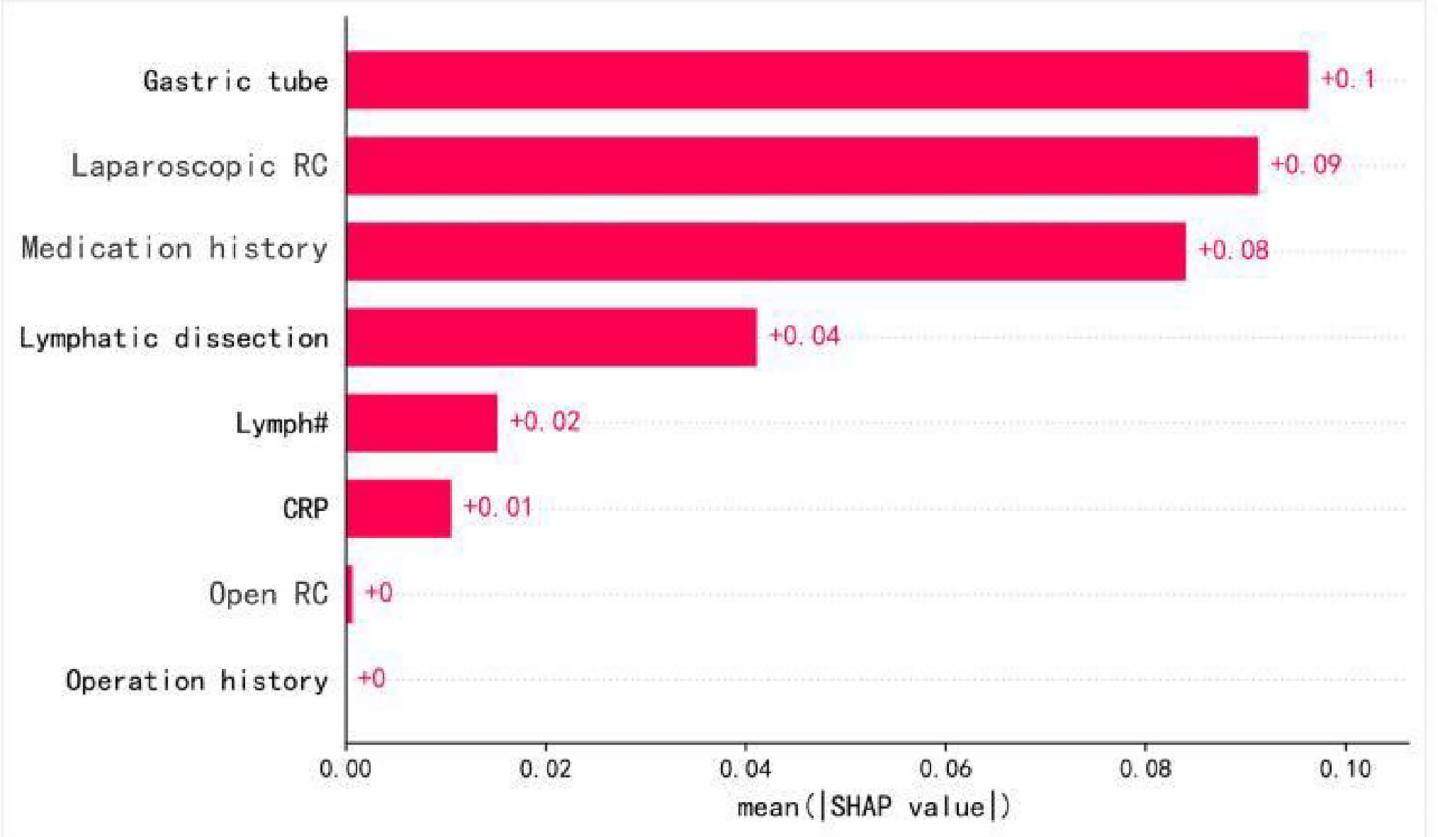
Feature importance ranking. SHAP-based feature importance ranking in the BPNN model. Higher-ranked features indicate greater contribution to POI prediction. ‘Gastric tube’ refers specifically to intraoperative nasogastric tube placement.

SHAP analysis was used to evaluate the importance of each feature in predicting POI (Figure 4). The analysis indicated that “Gastric tube” was the most significant predictive variable, with an average SHAP value of 0.09. This was followed by “Laparoscopic RC” and “Medication history” both of which had SHAP values of 0.08. “Lymphatic dissection” had a SHAP value of 0.04. “Lymph#” (lymphocyte count), “Intraoperative fluid intake,” and “CRP,” had SHAP values of 0.01, indicating a smaller contribution to the model’s prediction.

### SHAP analysis results for key predictive features

**Figure 5:**
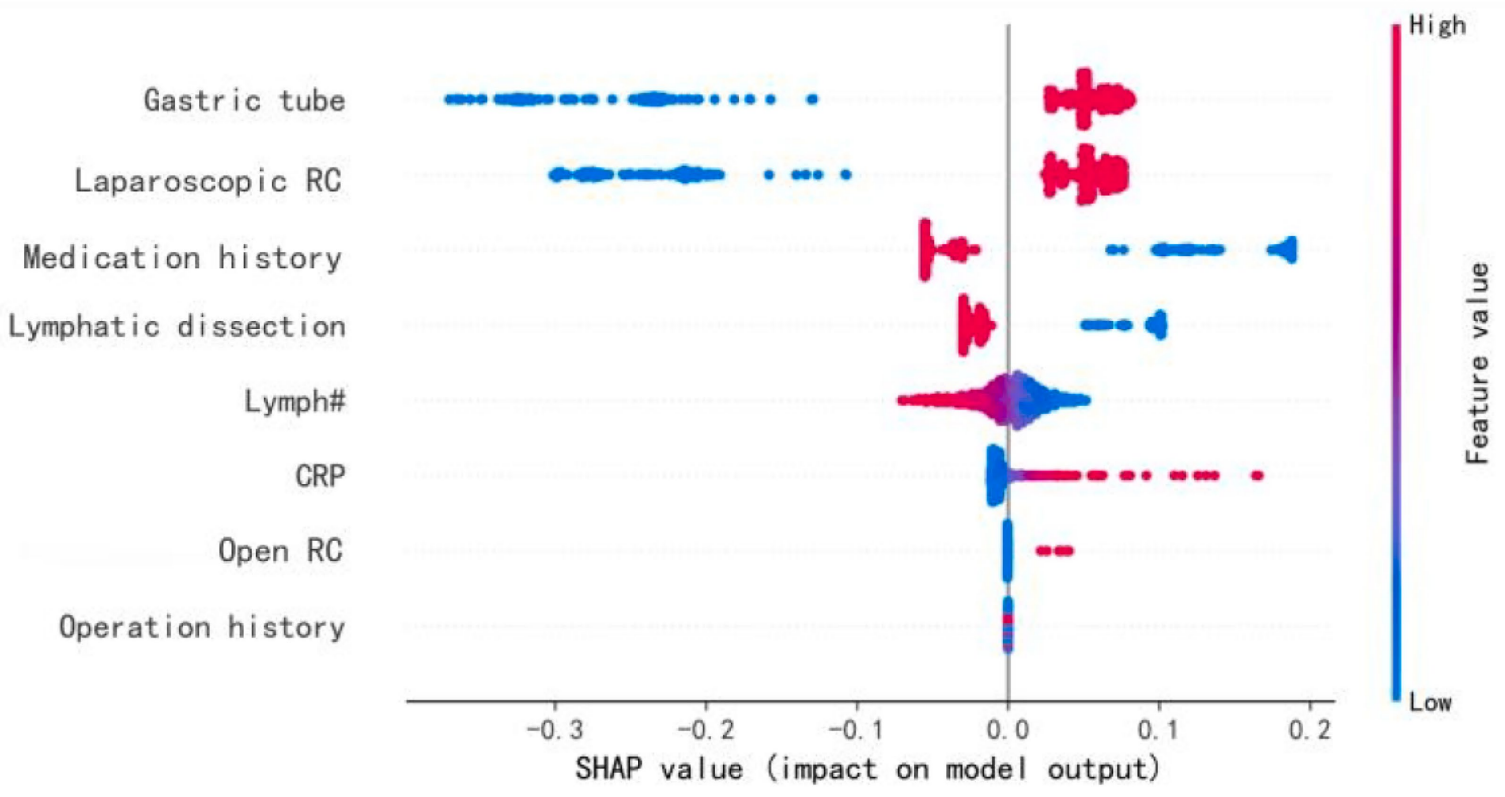
Characteristics of the selected model (BP neural network model): SHAP Value summary graph of top-12 variables and their impact on the prediction. “SHAP summary plot of the top 12 features influencing POI risk in the BPNN model. Red indicates positive contribution, blue indicates negative. ‘Gastric tube’ denotes intraoperative placement.

Figure 5 summarizes the SHAP values for the top 12 variables in the BPNN model. “Gastric tube” and “Laparoscopic RC” were the most influential features that significantly impacted POI prediction. “Medication history” and “Lymphatic dissection” also contributed notably. Other features, such as “Lymph#,” “Intraoperative fluid intake,” and “CRP,” had smaller impacts, while variables like “BMI” showed minimal influence. The SHAP value distribution provides insights into the most critical factors for predicting postoperative ileus.

### Interactive SHAP analysis of feature interactions

**Figure 6:**
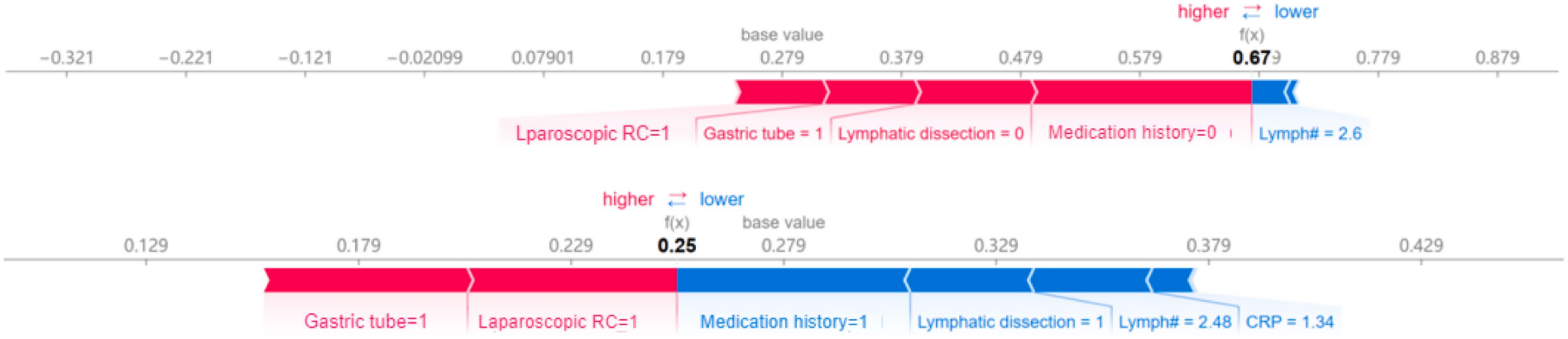
The chart visualises of interactive SHAP. Interactive SHAP plot for an individual prediction, showing feature contributions to POI risk. Red (right-pointing) arrows indicate higher risk; blue (left-pointing) arrows indicate lower risk. ‘Gastric tube’ refers to intraoperative placement.

The interactive SHAP analysis demonstrated how the specific values of key features influenced the model’s POI predictions (Figure 6). Positive contributors included “Laparoscopic RC = 1” (laparoscopic surgery), “Gastric tube = 1” (presence of a gastric tube), “Lymphatic dissection = 0” (no lymphatic dissection), and “Medication history = 0” (no medication history), all of which were associated with an increased predicted risk of POI. In contrast, negative contributors included “Medication history = 1,” “Lymphatic dissection = 1,” as well as lower CRP and higher lymphocyte values. For instance, a lymphocyte count greater than 2.48 × 10^9^/L or CRP lower than 1.34 mg/L was associated with reduced POI risk, reflecting model-derived inflection points rather than exact clinical thresholds. Notably, “Gastric tube = 1” and “Laparoscopic RC = 1” consistently exhibited strong positive SHAP values, while “Lymph# > 2.6” showed only a marginal negative effect on POI risk.

## Discussion

In a retrospective analysis of 1,062 patients who underwent RC over an 11-year period at a large medical center, we observed a 28.9% incidence of POI, which is consistent with a previous report (Grilo et al., 2024). We developed and internally validated an interpretable machine learning model (AUC = 0.828) that accurately predicted the risk of POI within 24 h after surgery. Compared to traditional regression-based nomograms, the machine learning model showed superior predictive performance by capturing complex nonlinear interactions among preoperative, intraoperative, and immediate postoperative variables. Key predictors identified via Shapley additive explanations included intraoperative nasogastric tube placement, surgical approach, medication history, lymph node dissection, lymphocyte count, and C-reactive protein level.

Our findings are consistent with those of previous studies in several key aspects while also providing novel insights. The observed POI incidence closely mirrors the findings of recent multicenter studies (Xue et al., 2021; Qi et al., 2022), supporting the external validity of our dataset. However, compared to previously developed nomograms, Xue et al. (2021) (AUC = 0.74) and Qi et al. (2022) (AUC = 0.76), our machine learning approach significantly improved the predictive accuracy, suggesting that nonlinear modeling enhances POI risk prediction following complex pelvic surgery. Similar performance improvements have been reported in machine learning studies on gastrointestinal surgery (Peters et al., 2017), further highlighting the broad applicability of these techniques in perioperative risk stratification.

Notably, SHAP-based model analysis provides new perspectives that challenge the conventional understanding. First, in our cohort, laparoscopic RC had a higher predictive weight for POI than open or robot-assisted RC, contrasting with the prevailing view that minimally invasive techniques generally promote faster recovery (Rockall et al., 2014; Tanneru et al., 2021). This discrepancy may stem from limited instrument dexterity owing to the absence of robotic wrist articulation, prolonged pneumoperitoneum, and increased procedural complexity, which are often underrepresented or diluted in standard regression models. Second, intraoperative nasogastric tube placement was the strongest predictor. Although the ERAS guidelines discourage routine nasogastric intubation and promote early enteral nutrition (Feng et al., 2022), RC involves prolonged pelvic procedures and urinary diversion, prompting surgeons to make real-time decisions regarding prophylactic placement. In our study, nasogastric tube use functioned as a “real-time flag” by the surgical team to indicate high-risk procedures, an intraoperative signal automatically captured by the model, underscoring its potential as an actionable risk indicator in the post-anesthesia care unit. Moreover, the unexpected association between omission of concurrent lymph node dissection and increased POI risk may reflect underlying differences in surgical technique, surgeon experience, or pelvic exposure—subtle signals often overlooked in traditional studies focused on average treatment effects (Xue et al., 2021; Fang et al., 2022).

A key strength of our machine learning model is its ease of integration into existing hospital workflow. All identified predictors are readily available in standard perioperative records, enabling the seamless integration of the model into electronic health record systems without additional data collection. Intraoperative nasogastric tube placement may serve as an early postoperative indicator for identifying patients who may benefit from enhanced gastrointestinal monitoring. Moreover, elevated CRP levels and decreased lymphocyte counts, which are established markers of systemic inflammation and immunosuppression (Peters et al., 2017; Tang et al., 2020), are associated with delayed gastrointestinal recovery. These routinely available laboratory parameters may inform personalized postoperative strategies, including tailored fluid management, nutritional support, and proactive monitoring of complications.

Additionally, our findings underscore the need for individualized postoperative care pathways, challenging the “one-size-fits-all” approach that is commonly embedded in standardized perioperative protocols. Specifically, laparoscopic RC may require distinct postoperative management strategies compared to open or robot-assisted procedures. Recognizing these differences allows clinicians to tailor care plans based on patient- and procedure-specific risk profiles, thereby improving the postoperative outcomes. Early real-time risk stratification may facilitate timely identification of high-risk patients and support critical decisions regarding patient placement, resource allocation, and monitoring intensity.

## Limitations and future directions

This study had several limitations. First, the retrospective, single-center design may limit the generalizability of the findings owing to institutional differences in surgical techniques, perioperative protocols, and patient demographics. Second, potentially influential variables, such as cumulative opioid dosage and detailed medication regimens, were unavailable owing to the constraints of retrospective data, limiting our ability to fully elucidate the underlying causal mechanisms. Finally, the model has not yet been externally validated in an independent cohort, leaving uncertainties regarding its broader applicability and portability.

Future studies should address these limitations through prospective multicenter validation to assess the robustness and generalizability of the machine learning model. Additionally, implementation trials are needed to assess the impact of real-time risk alerts integrated into electronic health records, clinical outcomes, resource use, and recovery trajectories. These efforts will enhance clinical adoption and provide a strong evidence base for the broader integration of machine learning–based predictive tools into routine perioperative care.

## Conclusion

This study developed an interpretable machine learning model based on routinely available perioperative data to accurately predict the occurrence of POI within 24 h in patients undergoing RC. Our model outperformed existing predictive approaches and identified novel clinically actionable predictors. Future external validation and integration of the model into electronic clinical workflows may support informed clinical decision-making and enable personalized gastrointestinal recovery strategies, representing a critical milestone toward the data-driven optimization of perioperative care.

## Data Availability

The data analyzed in this study is subject to the following licenses/restrictions: the datasets comprise patient-level electronic medical records containing protected health information and cannot be shared publicly. De-identified data may be made available upon reasonable request to the corresponding author, subject to Institutional Review Board approval and a data-use agreement. All summary statistics, tables/figures, and the analysis scripts/notebooks are provided in the article and Supplementary material. Requests to access these datasets should be directed to chenxp@sysucc.org.cn.
